# Genetic mapping of the *Fusarium* head blight resistance gene in wheat Guixie 3

**DOI:** 10.1007/s13353-025-00972-6

**Published:** 2025-05-15

**Authors:** Yonglu Luo, Bin Cheng, Tianqing Chen, Jianshu Sui, Wenqiang Wu, Qing Xu, Wei Wang

**Affiliations:** 1 Guizhou Institute of Upland Crops, Guizhou Academy of Agricultural Sciences, Guiyang, 550006 Guizhou People’s Republic of China; 2 Grain Crop Research Institute, Hubei Academy of Agricultural Sciences, Wuhan, 430070 Hubei People’s Republic of China

**Keywords:** Common wheat, *Fusarium* head blight, 55 K SNP array, Genetic mapping, Candidate genes

## Abstract

*Fusarium* head blight (FHB) is a global detrimental disease affecting wheat production. While Guixie 3 shows strong resistance to FHB, its resistance mechanism is not well understood. Hence, this study aims to elucidate the genetic basis of disease resistance in Guixie 3 and identify new genetic resources for FHB resistance in wheat. The study used an *F*_2:7_ recombinant inbred line population developed by crossing Avocet S with Guixie 3. FHB resistance was phenotypically evaluated across 2 years and two locations (i.e., four environments) after single-floret inoculation, and it was genetically mapped using the wheat 55 K single-nucleotide polymorphism array. A total of 15 quantitative trait loci (QTLs) for FHB resistance were detected on chromosomes 1D (2), 2A (2), 2B (3), 2D.1 (2), 3B (1), 4A (1), 4B (1), 4D (1), 5A (1), and 5B.2 (1). Notably, a QTL on chromosome 2D.1, designated as *Qfhb.gaas.2D.1–1*, was consistently detected in two environments. This QTL spanned the interval AX-86163393 to AX-110072786, with a genetic interval of 45.12–46.51 cM and a physical interval of 35.68–37.04 Mb (1.36 Mb). It explained 14.07–33.00% of the phenotypic variation. Furthermore, 39 candidate genes were identified in this target region, of which eight were predicted to be associated with FHB resistance. These candidate genes will be further analyzed and validated for FHB resistance in future studies.

## Introduction

*Fusarium* head blight (FHB) caused by *Fusarium graminearum* is a disease that infects wheat (*Triticum aestivum* L.) heads, posing a significant threat to wheat yield. FHB also contaminates food supplies with its mycotoxin, deoxynivalenol (DON), which is toxic to humans and animals. FHB incidence in China has increased, with large-scale outbreaks exceeding 5.5 million ha, spreading to the Huang-Huai wheat-growing region (Chen et al. 2012; Zhang et al. 2018; Huang et al. 2020). To tackle this challenge, breeding and promoting disease-resistant wheat varieties have been recognized as economical environmental strategies for controlling FHB. Molecular breeding approaches, such as identifying quantitative trait loci (QTLs) or genes for FHB resistance and pyramiding multiple resistance genes, have become crucial in addressing the problem and ensuring food security and public health.

FHB resistance in wheat is a quantitative trait controlled by multiple genes, and it is often influenced by environmental factors (e.g., temperature and humidity) and agronomic characteristics (e.g., plant height and heading date). By systematically evaluating FHB resistance, agricultural scientists have successfully identified and developed numerous high-resistance wheat varieties (He et al. 2014). The most commonly used FHB-resistant resources are the Chinese varieties Sumai 3 and Wangshuibai. Over 600 genetic loci associated with FHB resistance have been identified on the 21 wheat chromosomes, offering valuable resources for breeding efforts (Steiner et al. 2017; Buerstmayr et al. 2020; Liu et al. 2009; Ma et al. 2020). In addition, various studies have mapped FHB resistance QTLs on different chromosomes, contributing to the understanding and improvement of FHB resistance in wheat. For example, Zhang et al. (2014) mapped FHB resistance QTLs on chromosomes 3 A and 5 A in a recombinant inbred line (RIL) population derived from emmer wheat PI41025 and durum wheat Ben. Xinyao et al. (2016) located a QTL on chromosome 2D in a doubled haploid population derived from synthetic wheat (SYN1 × Ocoroni), with phenotypic variation explanation (PVE) of 25%. Prat et al. (2017) identified the *Fhb1* gene in Sumai 3-derived material (DBC-480), providing strong support for improving FHB resistance in durum wheat. In addition to well-known genes such as *Fhb1* and *Fhb2* from Sumai 3 and *Fhb7* from *Thinopyrum elongatum*, other resistance genes have been fine-mapped by developing large populations. These include *Fhb3* from *Elymus*; *Fhb4*, *Fhb5*, and *Fhb8* from Wangshuibai; *Fhb6* from *Leymus racemosus*; and *Fhb9* from Shi4185 (Liu et al. 2006; Cuthbert et al. 2006, 2007; Qi et al. 2008; Xue et al. 2010, 2011; Cainong et al. 2015; Guo et al. 2015; Wang et al. 2024; Zhang et al. 2024). Another wheat gene with FHB resistance potential is *TGA2* (Su et al. 2018); it enhances resistance by various mechanisms.

Upon infection by *F. graminearum*, defense-related genes, such as those encoding *β*−1,3-glucanase, chitinase, and thaumatin-like protein, are upregulated, providing potential applications for disease resistance (Pritsch et al. 2000, 2001). In addition, salicylic acid signaling plays a vital role in enhancing FHB resistance in wheat. Genes of the NPR1 family, particularly those located on the long arms of chromosomes 2 A and 2D, are closely associated with FHB resistance in winter wheat (Makandar et al. 2006; Gao et al. 2013; Yang et al. 2013; Yu et al. 2017; Diethelm et al. 2014). Other genes that contribute to FHB resistance include the isochorismate synthase gene in barley (Hao et al. 2018) and the TaWRKY70 (also known as TaWRKY45) (Kage et al. 2017) and phenylalanine ammonia lyase genes in wheat (Yu et al. 2015). Despite these advances, understanding of the mechanisms underlying FHB resistance remains limited. Therefore, the key tasks of the present study were to identify FHB resistance QTLs or genes, develop functional markers, and evaluate their utility in breeding.

Third-generation molecular markers, such as single-nucleotide polymorphism (SNP) markers, offer high density, genetic stability, and rich polymorphism. The wheat 55 K SNP genotyping array contains approximately 55,000 SNP markers evenly distributed across the 21 wheat chromosomes. Compared with similar arrays, it offers higher effective marker ratios, complete genomic information for downstream analyses, and cost efficiency. This array has become a powerful tool for high-density linkage analysis and rapid gene discovery on a genome-wide scale. Two prominent FHB resistance studies using SNP arrays include Burt et al. (2015), who mapped *QFhs.jic-4 AS*, a resistance gene derived from *T. aestivum* subsp. *macha*, using a 5 K SNP array, and He et al. (2016), who mapped stable QTLs on chromosome 2DL in a CIMMYT-derived bread wheat population. These QTLs explained 15–22% of the phenotypic variation across multiple environments. By using SNP arrays, researchers can accurately locate QTLs, obtain closely linked molecular markers, and assess their breeding efficacy. Marker-assisted selection can then accelerate the development of new FHB-resistant wheat varieties.

Guixie 3 was developed as a hybrid of wild emmer wheat (*T. dicoccoides*) and wild oat (*Avena fatua*) backcrossed with the wheat variety Guinong 22. Cytological studies have confirmed that it is a common wheat variety with 42 chromosomes (Geng et al. 2009). In 2013–2014, Guixie 3 exhibited FHB resistance comparable to Sumai 3 under four environments (Xiao et al. 2016).

This study used a RIL population derived from a cross between the susceptible variety Avocet S and the resistant variety Guixie 3. High-throughput genotyping with the wheat 55 K SNP array was performed on the RIL population, followed by precise phenotypic evaluation under four conditions (i.e., 2 years and two locations) after single-floret inoculation. QTLs were mapped using interval mapping techniques, and candidate genes in stable QTL regions were identified for further cloning and functional validation in the future.

## Materials and methods

### Experimental materials

Avocet S, sourced from Australia, lacks any disease resistance genes. The population resulting from its hybridization with Guixie 3 maintains a homogeneous genetic background, rendering it ideally suited for genetic research. Hence, in this study, the female parent was the susceptible wheat variety Avocet S, while the male parent was Guixie 3, a resistant line preserved in the Guizhou Academy of Agricultural Sciences, Guiyang, China. These two parents were crossed and self-pollinated to obtain the *F*_2_ generation. Subsequently, *F*_2:7_ RILs (228 lines) were developed using the single-seed descent method for field phenotyping and genotyping of FHB resistance. Highly resistant Sumai 3, moderately resistant Yangmai 158, moderately susceptible Huamai 20, and highly susceptible Aikang 58 were included as experimental controls.

The highly pathogenic FHB strain “Huanggang 1” preserved in the Plant Protection and Soil Fertilizer Institute of the Hubei Academy of Agricultural Sciences, Hubei, China, was used for FHB resistance evaluations.

### Field experiments

Field experiments were conducted during the growing seasons of 2023–2024 at two sites with yellow soil: (1) the Wheat Experimental Base of the Guizhou Academy of Agricultural Sciences, Guiyang, China (GY; 26°30′ N, 106°39′ E; altitude 1154 m); and (2) the Ezhou Experimental Base of the Hubei Academy of Agricultural Sciences, Hubei, China (HB; 30°53′ N, 114°39′ E; altitude 47.9 m). The four experimental environments were designated as 2023GY, 2024GY, 2023HB, and 2024HB. From inoculation to the full onset of disease (March–May), the average rainfall for 2023GY was 81.33 mm, with an average temperature of 17.36 °C; for 2024GY, the average rainfall was 69.80 mm, with an average temperature of 22.53 °C; for 2023HB, the average rainfall and average temperature were 263.07 mm and 18.17 ℃, respectively; for 2024HB, the average rainfall and average temperature were 32.67 mm and 19.00 ℃, respectively. (The meteorological data is sourced from the China Meteorological Data Network.)

Each variety was planted in two rows measuring 1 m, with 30-cm spacing between rows. Fifty seeds were sown in each row. The experiments were performed in two replicates. Field management followed standard breeding practices.

#### Preparation of spore suspension for “Huanggang No. 1”

The production of the “Huanggang No. 1” spore suspension was undertaken at the Dry Crop Research Institute of Guizhou Academy of Agricultural Sciences and the Grain Crop Research Institute of Hubei Academy of Agricultural Sciences.

Potato dextrose agar (PDA) medium preparation: Peeled potatoes (200 g) were weighed, cut into small pieces with a knife, and placed in a pot. Subsequently, 1 L of deionized water was added and boiled for 30 min. Next, 20 g of glucose and 15 g of agar powder were taken in a 2-L beaker, and an appropriate amount of distilled water was added. The boiled potatoes were filtered with gauze to remove the residue, and the filtrate was transferred to the beaker from the previous step. Distilled water was added to make up to 1 L. After sterilization at 121 °C for 25 min in an autoclave, the filtrate was dried and transferred to a clean bench for use.

Mung bean broth preparation: Mung beans (30 g) were boiled in 1 L of distilled water and cooked at 100 °C for 10 min. After cooking, the mung bean residue was filtered with gauze, and the filtrate was retained. The filtrate was sterilized in an autoclave at 121 °C for 20 min and stored at room temperature for future use.

Conidial inoculum preparation: About 20 days before inoculation, the “Huanggang No. 1” strain stored in a 4 °C refrigerator was transferred to a fresh PDA medium plate for activation, and the mycelium was allowed to cover the plate for later use. The activated mycelium was picked and inoculated into 3% mung bean broth, which was placed in an oscillating incubator (25 °C, 180 rpm) and incubated for 7 days. Microscopic examination was conducted to confirm the production of a large number of spores. After filtration through sterile gauze, the filtrate was collected. The spores were counted using a hemocytometer and mixed, and the spore suspension was diluted in the filtrate to a concentration of 1 × 10^5^ spores/mL and stored in a 4 °C refrigerator for later use.

### Phenotypic evaluation of FHB resistance at the adult stage

A spore suspension of “Huanggang 1” was cultured at a concentration of 1 × 10^5^ spores/mL. At the early flowering stage, 10 µL of the suspension was injected into the central floret of randomly selected spikes (10 spikes per row, 20 spikes of each variety). Inoculated spikes were marked by clipping the awns. After inoculation, spikes were enclosed in plastic bags to maintain humidity for 72 h, followed by misting. Disease symptoms were evaluated 21 days after inoculation based on the infected spikelet rate, calculated as infected spikelet rate = number of infected spikelets per spike/total number of spikelets per spike.

### DNA extraction and 55 K SNP genotyping

At the one-leaf, one-heart seedling stage, genomic DNA was extracted from the RIL population and parental lines using a plant genomic DNA extraction kit (DP320 DNAsecure Plant Kit; Tiangen Biotech, Beijing, China). DNA quality was verified using 1% agarose gel electrophoresis and a GelDoxXR system (Bio-Rad Laboratories, Hercules, CA, USA). Qualified samples were genotyped using the wheat 55 K SNP array, which was performed by China Golden Marker Company (Beijing, China). Quality control (QC) standards were based on detection rate, minor allele frequency (MAF), and heterozygosity. Sample QC requirements were dish quality control value > 0.82, detection rate ≥ 85%, and heterozygosity ≤ 10%, while marker QC requirements were poly high resolution, detection rate ≥ 90%, MAF ≥ 4%, heterozygosity ≤ 10%, and only biallelic loci. Homozygous loci with polymorphisms between parents were retained for subsequent analyses.

### QTL mapping

The genetic map was constructed using the Kosambi function in JoinMap v.4.0, covering the 21 wheat chromosomes with a total length of 4363.9 cM and a marker density of 1.5 cM (Gao et al. 2022). QTLs were mapped with QTL IciMapping v.4.0 (https://isbreedingen.caas.cn/software/qtllcimapping/294607.htm) using the ICIM-ADD method. Parameters included a 0.1 cM step length and a regression probability threshold of *P* < 0.001. QTLs with a logarithm of the odds (LOD) score > 2.5 were detected, and their genetic contribution rates and additive effects were calculated. Detected QTLs were named in the format: “Q + trait abbreviation + institution abbreviation + chromosome number + locus order.”

### Prediction and analysis of candidate genes

The physical positions of flanking markers for the detected QTLs were queried, and candidate gene information was retrieved by aligning with the Chinese Spring reference genome (v.2.0; International Wheat Genome Sequencing Consortium). Additional details were obtained from the Wheat Omics database (Ma et al. 2021).

### Data analysis

Phenotypic data analyses, including calculation of means, standard deviations, coefficients of variation (CV), normality tests, one-way analysis of variance (ANOVA), and correlation analysis, were performed using Microsoft Excel 2021 (Microsoft Corp., Redmond, WA, USA) and SPSS v.21.0 (SPSS Inc., Chicago, IL, USA). Normality plots were generated with Origin 2019b (OriginLab Corp., Northampton, MA, USA). Broad-sense heritability (*H*^2^) was calculated using ANOVA in QTL IciMapping. *H*^2^ = *V*_G_/(*V*_G_ + *V*_E_), where *V*_G_ is genetic variance and *V*_E_ is environmental variance. QTL peaks were visualized using ggplot2 (https://ggplot2.tidyverse.org/) in R and MapChart v.2.32 (https://www.wur.nl/en/show/Mapchart.htm).

## Results

### Phenotypic evaluation of FHB resistance

**Fig. 1:**
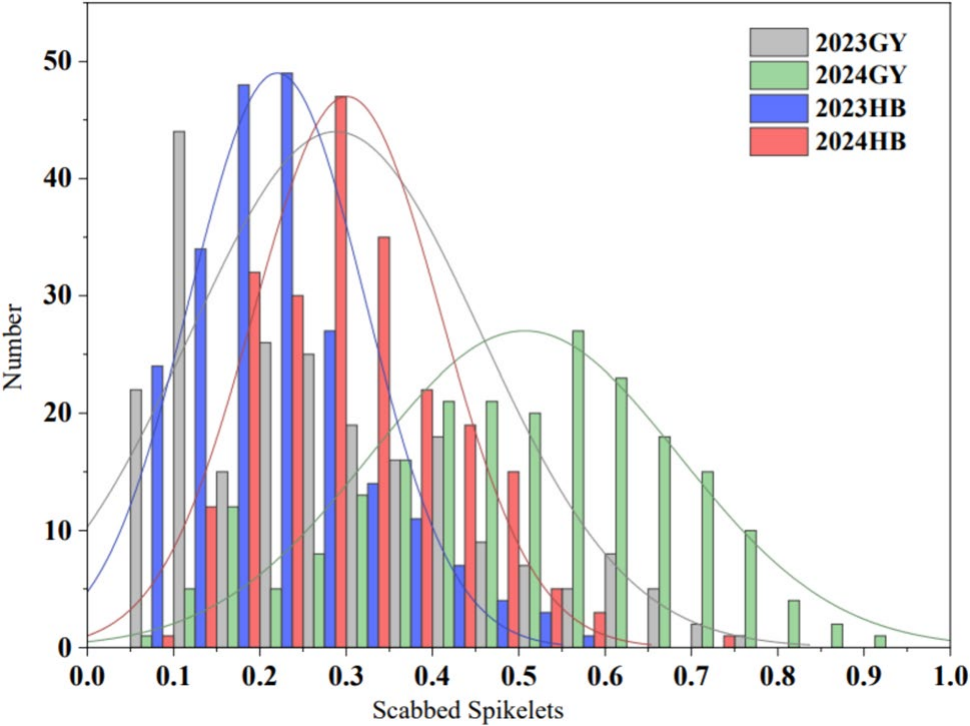
Phenotypic distribution for the percentage of infected spikelets

Based on the infected spikelet rate measured in the parents Guixie 3 and Avocet S, the RIL population across the four environments showed significant variation in resistance (Table 1), with infected spikelet rates ranging from 0.06 to 0.97 and CV between 35.52 and 58.69%. In addition, the skewness and kurtosis of the infected spikelet rates were less than 1 in absolute value across all environments. The data displayed a continuous, near-normal distribution. These characteristics align with the genetic nature of quantitative traits, making the population suitable for QTL mapping analysis (Fig. 1).

**Table 1:** Description of the percentage of infected spikelets for parents and RILs

Environment	Parental strain		RILs						
	GX3	AvS	Max	Min	Mean	SD	CV (%)	Skewness	Kurtosis
2024GY	0.20	0.70	0.92	0.09	0.51	0.18	35.52	−0.263	−0.479
2024HB	0.31	0.68	0.70	0.08	0.30	0.11	36.23	0.568	0.134
2023GY	0.09	0.71	0.79	0.06	0.29	0.17	58.69	0.737	−0.228
2023HB	0.17	0.58	0.97	0.06	0.22	0.10	46.28	0.966	0.894

2024GY: 2024 Guiyang, 2024HB: 2024 Hubei, 2023GY: 2023 Guiyang, 2023HB: 2023 Hubei

Variance analysis revealed that the environment has a significant impact on FHB occurrence (Table 2). The *H*^2^ of the infected spikelet rate was 69.63%, indicating that phenotypic differences were primarily determined by genetic factors, with minimal environmental influence. Correlation coefficients among the four environments ranged from 0.112 to 0.505. Apart from a low correlation between 2023HB and 2023GY, infected spikelet rates in the other environments were significantly positively correlated. Severe lodging at the Hubei Ezhou Experimental Base in 2023 might have contributed to lower or insignificant correlations between years (Table 3).

**Table 2:** ANOVA for the phenotypic data and broad heritability of FHB in the RIL population under four environments

Variation source	Sum of squares	Df	Mean square	*F* value	Significance
Inter group	10.19	3	3.3962	163.976	0.0001
Intra group	18.31	884	0.0207		
Total	28.50	887			
*H*^2^%	69.63

**Table 3:** Correlation analysis for the percentage of infected spikelets

Environment	2024GY	2024HB	2023GY	2023HB
2024GY	1.000			
2024HB	0.447**	1.000		
2023GY	0.505**	0.298**	1.000	
2023HB	0.142*	0.176**	0.112	1.000

2024GY: 2024 Guiyang, 2024HB: 2024 Hubei, 2023GY: 2023 Guiyang, 2023HB: 2023 Hubei^*^At the 0.05 level (two-tailed), the correlation is significant^**^At the 0.01 level (two-tailed), the correlation is significant

### QTL mapping

**Fig. 2:**
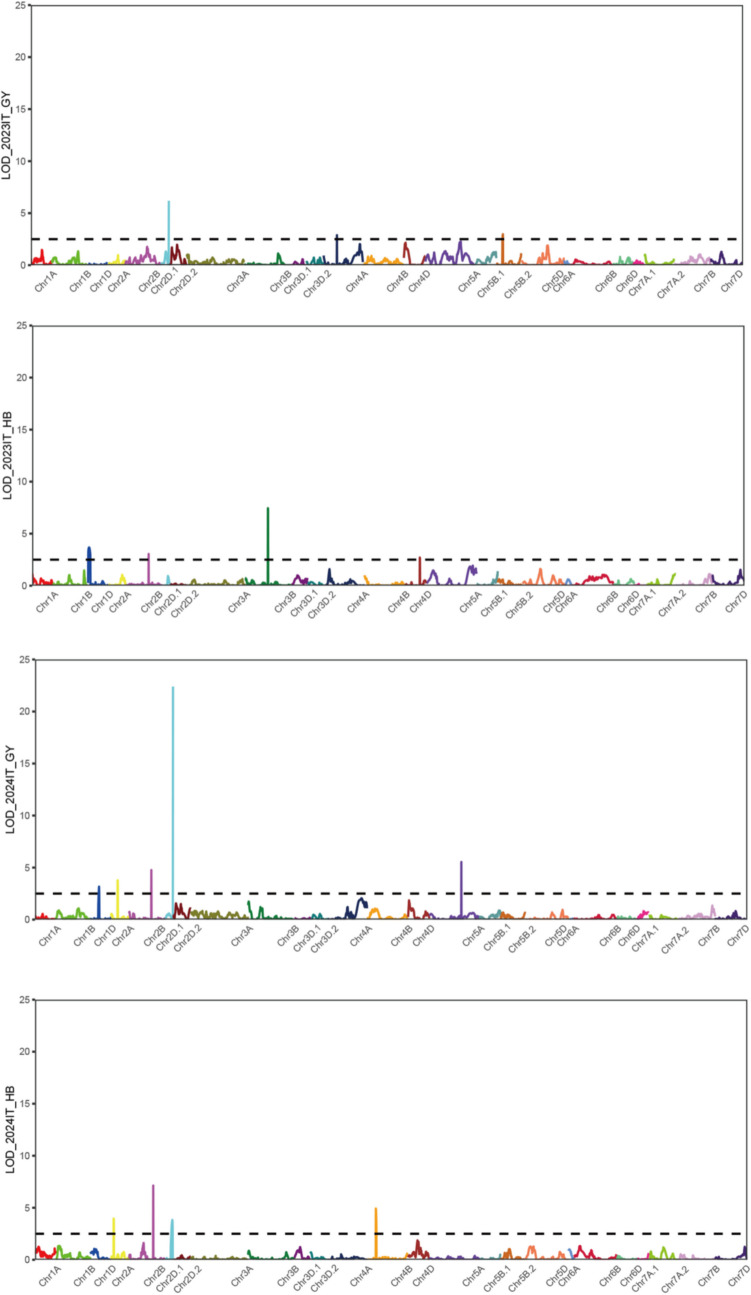
QTLs for FHB resistance located on all chromosomes in four different environments

**Fig. 3:**
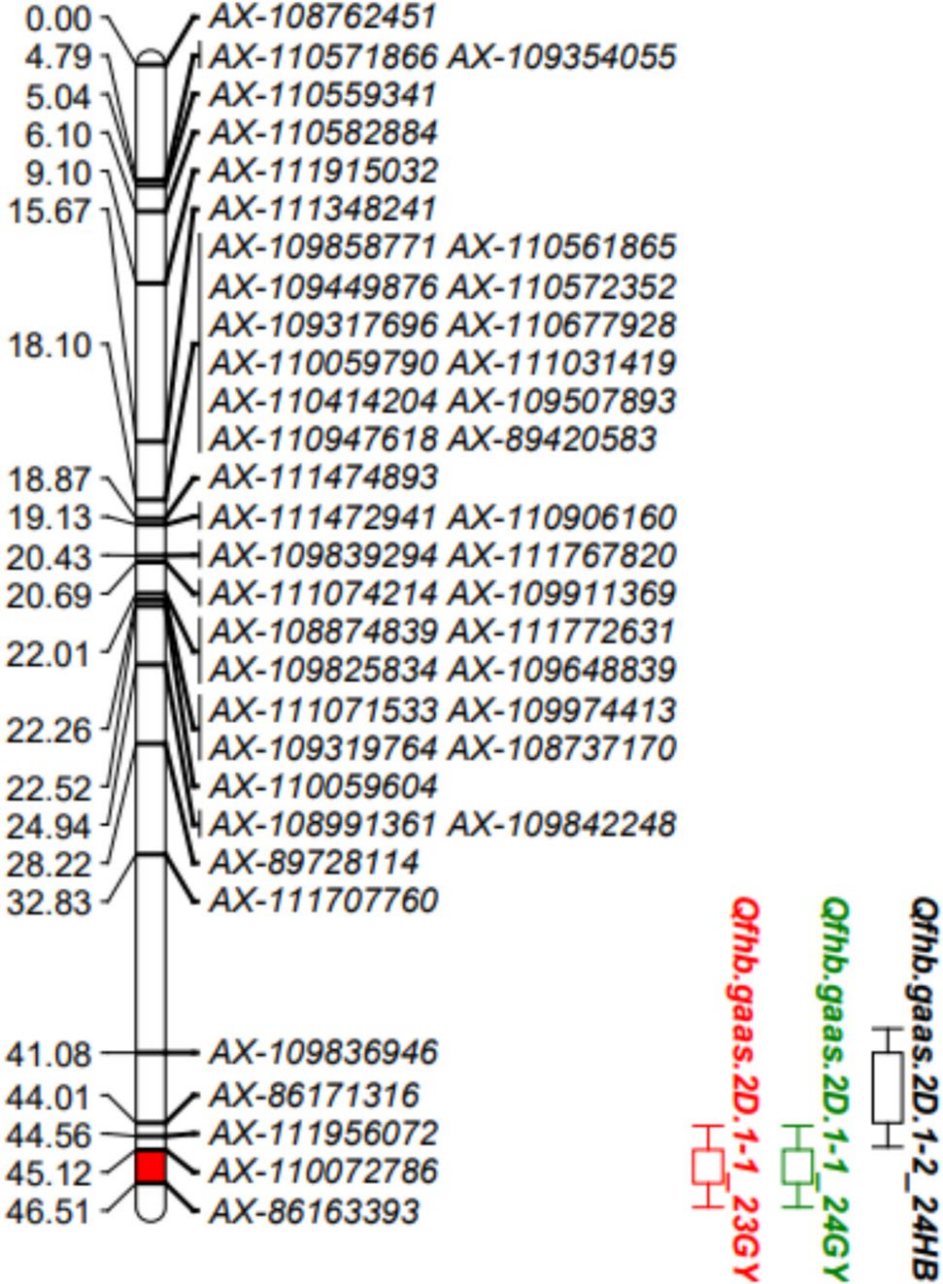
Important QTLs for FHB resistance located on chromosome *2D.1*

Using genotypic data from the wheat 55 K SNP array and phenotypic evaluation results of FHB resistance, QTLs were mapped with QTL IciMapping. A total of 15 QTLs for FHB resistance were detected on chromosomes 1D (2), 2 A (2), 2B (3), 2D.1 (2), 3B (1), 4 A (1), 4B (1), 4D (1), 5 A (1), and 5B.2 (1). Among these, the *Qfhb.gaas.2D.1–1* QTL was identified in two environments. It was located in the genetic interval of 45.12–46.51 cM on chromosome 2D.1, corresponding to a physical interval of 35.68–37.04 Mb (1.36 Mb). The LOD scores for this QTL ranged from 6.19 to 22.40 across 2 years, with PVE of 14.07–33.00%. This stable major-effect QTL was flanked by markers AX-86163393 and AX-110072786 (Table 4, Figs. 2 and 3).

**Table 4:** QTL analysis for FHB resistance

QTL	Environment	Marker interval	Confidence interval/cM	Genetic interval/cM	Physical interval/Mb	LOD	PVE (%)	Add
*Qfhb.gaas.1D-1*	2024GY	AX-109864616 ~ AX-109864377	45.5 ~ 49.5	43.62 ~ 48.23	335.59 ~ 372.86	3.17	3.69	− 0.03
*Qfhb.gaas.1D-2*	2023HB	AX-110957998 ~ AX-111548588	1.5 ~ 14.5	1.17 ~ 13.60	7.92 ~ 10.55	3.67	7.32	− 0.03
*Qfhb.gaas.2 A-1*	2024GY	AX-108752520 ~ AX-109958635	36.5 ~ 38.5	36.78 ~ 38.11	637.67 ~ 657.90	3.78	4.43	0.04
*Qfhb.gaas.2 A-2*	2024HB	AX-110557323 ~ AX-110564463	13.5 ~ 15.5	13.85 ~ 15.73	70.27 ~ 71.10	3.96	6.39	0.03
*Qfhb.gaas.2B-1*	2024GY	AX-110430167 ~ AX-108740839	128.5 ~ 129.5	128.86 ~ 129.50	642.71 ~ 643.29	4.76	5.64	0.04
*Qfhb.gaas.2B-2*	2023HB	AX-108740839 ~ AX-108954862	129.5 ~ 130.5	129.50 ~ 130.15	643.29 ~ 647.29	3.05	4.97	0.02
*Qfhb.gaas.2B-3*	2024HB	AX-110613560 ~ AX-109327254	139.5 ~ 140.5	139.27 ~ 140.04	697.45 ~ 712.79	7.13	11.83	0.04
*Qfhb.gaas.2D.1–1*	2023GY	AX-86163393 ~ AX-110072786	45.5 ~ 46.0	45.12 ~ 46.51	35.68 ~ 37.04	6.19	14.07	− 0.06
	2024GY	AX-86163393 ~ AX-110072786	45.5 ~ 46.0	45.12 ~ 46.51	35.68 ~ 37.04	22.40	33.00	− 0.10
*Qfhb.gaas.2D.1–2*	2024HB	AX-109836946 ~ AX-86171316	36.5 ~ 44.5	41.08 ~ 44.01	32.80 ~ 34.23	3.83	6.55	− 0.03
*Qfhb.gaas.3B*	2023HB	AX-110432985 ~ AX-110561047	130.5 ~ 132.5	130.48 ~ 131.13	584.53 ~ 584.95	7.45	12.89	0.04
*Qfhb.gaas.4 A*	2023GY	AX-109896933 ~ AX-111229802	76.5 ~ 77.5	76.69 ~ 77.33	567.82 ~ 571.72	2.88	6.11	− 0.04
*Qfhb.gaas.4B*	2024HB	AX-111162353 ~ AX-109018264	49.5 ~ 52.5	49.03 ~ 50.16	19.74 ~ 20.61	4.93	8.01	− 0.03
*Qfhb.gaas.4D*	2023HB	AX-110500045 ~ AX-89515151	84.5 ~ 86.5	84.40 ~ 85.49	426.52 ~ 432.77	2.70	4.45	0.02
*Qfhb.gaas.5 A*	2024GY	AX-110996595 ~ AX-110010456	183.5 ~ 184.5	183.66 ~ 184.70	591.46 ~ 592.42	5.54	6.54	− 0.05
*Qfhb.gaas.5B.2*	2023GY	AX-111212169 ~ AX-109941615	33.5 ~ 37.5	33.24 ~ 37.04	566.26 ~ 571.98	2.97	6.21	− 0.04

Positive additive effect indicates that increased resistance to disease was contributed by the AvS allele; negative by the GX3 allele*LOD* logarithm of odds score, *PVE* percentage of phenotypic variance explained by individual QTL, *Add* additive effect of the resistance allele

QTLs such as *Qfhb.gaas.4 A* and *Qfhb.gaas.5B.2* were detected only in 2023GY (LOD = 2.88–2.97, PVE = 6.11–6.21%). Similarly, *Qfhb.gaas.1D-1*, *Qfhb.gaas.2 A-1*, *Qfhb.gaas.2B-1*, and *Qfhb.gaas.5 A* were detected only in 2024GY (LOD = 3.17–5.54, PVE = 3.69–6.54%). Other QTLs, including *Qfhb.gaas.1D-2*, *Qfhb.gaas.2B-2*, *Qfhb.gaas.3B*, and *Qfhb.gaas.4D*, were specific to 2023HB (LOD = 2.70–7.45, PVE = 4.45–12.89%). In addition, *Qfhb.gaas.2 A-2*, *Qfhb.gaas.2B-3*, *Qfhb.gaas.2D.1–2*, and *Qfhb.gaas.4B* were detected only in 2024HB (LOD = 3.83–7.13, PVE = 6.39–11.83%).

### Functional annotation and candidate gene prediction of major QTLs

Based on the results, the *Qfhb.gaas.2D.1–1* QTL was mapped to the 35.68–37.04 Mb interval on chromosome 2D.1. Using the Wheat Omics database, the physical positions of flanking markers and candidate gene information were analyzed by aligning with the Chinese Spring reference genome. A total of 39 high-confidence genes with functional annotations were identified in this region. Among them, eight genes were predicted to be associated with disease resistance (Table 5): *TraesCS2D02G084600* encoding rho-type GTPase-activating protein 1 (RGA1); *TraesCS2D02G084700* encoding protein recognition of *Peronospora parasitica* 13 (RPP13); *TraesCS2D02G079200* encoding serine/threonine protein kinase; *TraesCS2D02G080000* encoding ascorbate peroxidase (APX); *TraesCS2D02G080400* and *TraesCS2D02G080500* encoding glucuronosyltransferase; and *TraesCS2D02G081900* and *TraesCS2D02G082100* encoding peroxidase (POD). Functional analysis of these candidate genes is needed to confirm their roles in FHB resistance.

**Table 5:** Functional annotation of candidate genes in important QTL intervals of *Qfhb.gaas.2D.1–1*

Candidate gene	Genomic location	Protein annotation
*TraesCS2D02G079200*	chr2D:33,903,808–33907017(−)	Serine/threonine-protein kinase PBL27
*TraesCS2D02G080000*	chr2D:34,227,832–34,231,931(+)	l-Ascorbate peroxidase 2, cytosolic
*TraesCS2D02G080400*	chr2D:34,329,547–34,332,204(+)	Probable glucuronosyltransferase Os03 g0287800
*TraesCS2D02G080500*	chr2D:34,341,730–34343945(−)	Probable glucuronosyltransferase Os03 g0287800
*TraesCS2D02G081900*	chr2D:35,240,707–35242242(−)	Peroxidase 21
*TraesCS2D02G082100*	chr2D:35,315,022–35316430(+)	Peroxidase 21
*TraesCS2D02G084600*	chr2D:36,657,312–36,661,415(+)	Putative disease resistance protein RGA1
*TraesCS2D02G084700*	chr2D:36,666,231–36,671,235(−)	Putative disease resistance RPP13-like protein 1
*TraesCS2D02G079000*	chr2D:33,878,259–33,878,715(+)	Ribosome production factor 2 homolog
*TraesCS2D02G079100*	chr2D:33,891,999–33,894,677(+)	Protein IRON-RELATED TRANSCRIPTION FACTOR 3
*TraesCS2D02G079300*	chr2D:33,934,810–33935341(+)	NAD(P)H-quinone oxidoreductase subunit 6, chloroplastic
*TraesCS2D02G079400*	chr2D:33,935,554–33,935,859(+)	NAD(P)H-quinone oxidoreductase subunit 4L, chloroplastic
*TraesCS2D02G079500*	chr2D:33,947,469–33,948,577(+)	NEP1-interacting protein-like 1
*TraesCS2D02G079600*	chr2D:33,952,048–33956269(−)	Two-component response regulator-like PRR37
*TraesCS2D02G080100*	chr2D:34,232,083–34234923(+)	Arogenate dehydratase/prephenate dehydratase 2, chloroplastic
*TraesCS2D02G080200*	chr2D:34,262,673–34,263,324(−)	TPD1 protein homolog 1 A
*TraesCS2D02G080300*	chr2D:34,272,506–34275681(−)	Protein PARTING DANCERS homolog
*TraesCS2D02G080800*	chr2D:34,891,820–34895411(+)	Elongation factor 1-alpha
*TraesCS2D02G080900*	chr2D:34,980,602–34984168(+)	Elongation factor 1-alpha
*TraesCS2D02G081000*	chr2D:34,988,005–34992393(−)	Probable metal-nicotianamine transporter YSL6
*TraesCS2D02G081200*	chr2D:34,994,756–34,998,921(−)	Probable metal-nicotianamine transporter YSL6
*TraesCS2D02G081400*	chr2D:34,999,904–35003326(−)	MLO-like protein 1
*TraesCS2D02G081500*	chr2D:35,022,527–35025791(−)	MLO-like protein 1
*TraesCS2D02G082000*	chr2D:35,259,471–35,263,395(+)	Phosphoinositide phospholipase C 2
*TraesCS2D02G082200*	chr2D:35,503,151–35513317(−)	Symplekin
*TraesCS2D02G082700*	chr2D:35,683,381–35,686,524(−)	Omega-3 fatty acid desaturase, chloroplastic
*TraesCS2D02G082900*	chr2D:35,791,185–35,801,312(−)	Syntaxin-binding protein 5
*TraesCS2D02G083000*	chr2D:35,803,955–35806291(+)	Increased DNA methylation 1
*TraesCS2D02G083600*	chr2D:36,112,676–36,114,025(+)	Salt stress–induced protein
*TraesCS2D02G083900*	chr2D:36,279,340–36284266(+)	26S proteasome regulatory subunit 6B homolog
*TraesCS2D02G084100*	chr2D:36,488,875–36,489,497(−)	Mitochondrial import inner membrane translocase subunit TIM17-2
*TraesCS2D02G084200*	chr2D:36,534,151–36,537,837(+)	Protein ESKIMO 1
*TraesCS2D02G084300*	chr2D:36,538,467–36,544,668(−)	Probable AMP deaminase
*TraesCS2D02G084400*	chr2D:36,576,473–36,578,292(+)	Probable protein phosphatase 2 C 32
*TraesCS2D02G084800*	chr2D:36,751,113–36,751,846(+)	BTB/POZ and MATH domain-containing protein 5
*TraesCS2D02G084900*	chr2D:36,771,092–36771628(+)	BTB/POZ and MATH domain-containing protein 3
*TraesCS2D02G085000*	chr2D:36,783,421–36,784,278(+)	BTB/POZ and MATH domain-containing protein 4
*TraesCS2D02G085100*	chr2D:36,789,337–36,800,416(−)	Importin-11
*TraesCS2D02G085500*	chr2D:37,020,758–37028300(−)	Isoflavone reductase homolog

## Discussion

### Genetic mapping of FHB resistance genes

FHB, a globally significant fungal disease, severely affects wheat yield and quality (Mesterházy 2006). FHB resistance–related QTLs have been identified on all 21 wheat chromosomes, with nine major loci (*Fhb1*–*Fhb9*) formally named. These loci are located on chromosomes 3BS, 6BS, 7Lr#1S, 4BL, 5 A, 1E terminal, 7E, 7D, and 2DL (Zhang et al. 2024). Among them, *Fhb9* was identified as a stable major QTL on the arm of chromosome 2DL using a RIL population derived from 4185 × Shijiazhuang 8. This QTL overlaps with *QFhb-2DL* in Ji5265, and it is flanked by SNP markers KASP-12056 (533.8 Mb) and KASP-525 (525.9 Mb), explaining 26.0–30.1% of the phenotypic variation (Zhang et al. 2024).

We used the 55 K SNP array to map the FHB resistance locus of Guixie 3 to chromosome 2D.1 and designated it as *Qfhb.gaas.2D.1–1*. The locus spans a physical interval of 35.68–37.04 Mb (1.36 Mb), with LOD scores ranging from 6.19 to 22.40 and PVE ranging from 14.07 to 33.00%. *Qfhb.gaas.2D.1–1* differs from the previously reported *Fhb9* locus, suggesting that they are not the same. It partially overlaps with a QTL on chromosome 2D (19.63–35.68 Mb, PVE = 4.98%) in an *F*_2:3_ population of Wangshuibai × Sy95-7 (Zhang et al. 2010). Similarly, *Qfhb.gaas.2D.1–1* partially overlaps with a QTL on chromosome 2D (34.23–35.68 Mb, PVE = 24.75%) in a Kenyon × 86ISMN2137 RIL population (McCartney et al. 2016). Thus, *Qfhb.gaas.2D.1–1* may represent the same QTL as these previously identified loci (Jia et al. 2005; Tamburic-Ilincic and Rosa 2019; Somers et al. 2003), but further validation is required. Notably, *Qfhb.gaas.2D.1–1* lies within a much smaller interval (1.36 Mb) than the intervals identified in previous studies (28.55, 60.88, and 16.45 Mb) (Jia et al. 2005; Tamburic-Ilincic and Rosa 2019; Somers et al. 2003), indicating higher mapping precision in this study. However, further research is needed to confirm and refine the position of this QTL.

Among the 15 FHB resistance loci identified, eight loci (*Qfhb.gaas.1D-1*, *Qfhb.gaas.1D-2*, *Qfhb.gaas.2D.1–1*, *Qfhb.gaas.2D.1–2*, *Qfhb.gaas.4 A*, *Qfhb.gaas.4B*, *Qfhb.gaas.5 A*, and *Qfhb.gaas.5B.2*) were derived from Guixie 3, and seven loci (*Qfhb.gaas.2 A-1*, *Qfhb.gaas.2 A-2*, *Qfhb.gaas.2B-1*, *Qfhb.gaas.2B-2*, *Qfhb.gaas.2B-3*, *Qfhb.gaas.3B*, and *Qfhb.gaas.4D*) were derived from the susceptible parent Avocet S. The PVE of loci from Avocet S was low (4.42–12.89%), illustrating the complexity of the inheritance of FHB resistance in wheat, as even susceptible materials may contain resistance loci.

Environmental conditions significantly influence FHB severity in wheat (Linkmeyer et al. 2016). Differences in environmental temperature and humidity may result in varying resistance performance of related genotypes, making QTL overlap challenging and minor-effect QTLs more apparent. For instance, *Qfhb.gaas.4 A* was detected only in the 2023GY experiment, *Qfhb.gaas.4B* only in the 2024HB experiment, and *Qfhb.gaas.5 A* only in the 2024GY experiment. These findings highlight the effect of environmental factors on FHB resistance in wheat. Accurate detection of minor-effect QTLs requires a large population as well as field experiments of multiple years and locations. QTL detection accuracy might have reduced because of the small size of the RIL population and limited experimental repetitions in this study. Future research should increase the genetic population, construct more refined genetic linkage maps, and accurately map the FHB resistance genes of Guixie 3.

### Identification of candidate genes

Genetic studies suggest that FHB resistance in wheat is controlled by both major- and minor-effect genes, making it highly sensitive to environmental fluctuations (Lin et al. 2007). Among the QTLs identified for FHB resistance, nine genes with confirmed effects have been reported, including the successfully cloned *Fhb1* and *Fhb7*. The *Fhb1* gene encodes a histidine-rich calcium-binding protein, but its resistance mechanism remains controversial (Su et al. 2019; Narumanchi et al. 2019). *Fhb7* encodes glutathione *S*-transferase that detoxifies DON by catalyzing its conjugation with glutathione (GSH) to form DON-GSH, providing a potential solution for mitigating mycotoxin contamination of food and feed (Wang et al. 2020).

We identified 39 candidate genes in the 35.68–37.04 Mb interval on chromosome 2D.1, which contains *Qfhb.gaas.2D.1–1*. Among these, eight genes were predicted to be associated with disease resistance and were candidate genes for *Qfhb.gaas.2D.1–1*: *TraesCS2D02G084600* encodes the disease resistance protein RGA1. *TraesCS2D02G084700* encodes the disease resistance protein RPP13. RGA proteins, containing conserved domains associated with disease resistance, are distributed throughout plant genomes and play critical roles in plant defense (Yi et al. 2002; Li et al. 2010; Zhou et al. 2004). RPP13 encodes products that trigger immune responses by recognizing pathogen effectors, functioning as a typical resistance gene (Rentel et al. 2008; Li et al. 2020a, b; Cheng et al. 2018). *TraesCS2D02G079200* encodes a serine/threonine protein kinase. *TraesCS2D02G080000* encodes APX. Resistance genes such as *Xa21* (rice), *Pto* (tomato), and *Lr10* (wheat) encode receptor-like protein kinases containing serine/threonine kinase domains (Song et al. 1995; Martin et al. 1993; Yang et al. 2001; Sun et al. 2004). APX also plays a key role in resistance by reducing hydrogen peroxide accumulation, enhancing jasmonic acid signaling, and improving resistance to various pathogens (Zhao 2023; Li et al. 2020a, b). *TraesCS2D02G080400* and *TraesCS2D02G080500* encode glucuronosyltransferase. *TraesCS2D02G081900* and *TraesCS2D02G082100* encode POD. UDP-glucosyltransferases convert DON to DON-3-glucoside, enhancing FHB resistance (He et al. 2018; Liu et al. 2020). POD, which regulates reactive oxygen species, may also contribute to resistance during pathogen infection (Fu et al. 2020).

Using the Avocet S × Guixie 3 RIL population and the 55 K SNP array, we mapped a FHB resistance QTL from Guixie 3. However, the QTL interval remains relatively large. Future work will focus on developing new markers, increasing the genetic population, and constructing secondary populations for fine mapping. By narrowing the target region, candidate genes can be identified and functionally validated, providing a solid foundation for FHB resistance breeding in wheat.

## Conclusions

Using the wheat 55 K SNP genotyping array and the RIL population derived from Avocet S × Guixie 3, 15 QTLs for FHB resistance were identified in four environments. Of these, one major QTL on chromosome 2D.1 (35.68–37.04 Mb) was consistently detected in multiple environments. Eight genes potentially associated with disease resistance were identified. These findings lay the foundation for further studies on molecular marker-assisted breeding and the functional identification of candidate genes.

## References

[CR1] Buerstmayr M, Steiner B, Buerstmayr H et al (2020) Breeding for Fusarium head blight resistance in wheat-progress and challenges. Plant Breeding 139:429–454

[CR2] Burt C, Steed A, Gosman N et al (2015) Mapping a type 1 FHB resistance on chromosome 4AS of *Triticum* macha and deployment in combination with two type 2 resistances. Theor Appl Genet 128:1725–1738 26040404 10.1007/s00122-015-2542-9 PMC4540761

[CR3] Cainong JC, Bockus WW, Feng Y et al (2015) Chromosome engineering, mapping, and transferring of resistance to Fusarium head blight disease from Elymus tsukushiensis into wheat. Theor Appl Genet 128:1019–1027 25726000 10.1007/s00122-015-2485-1

[CR4] Chen SH, Zhang Y, Bie TD et al (2012) Damage of wheat Fusarium head blight(FHB) epidemics and genetic improvement of wheat for scab resistance in China. Jiangsu Journal of Agricultural Sciences 28:938–942 (in Chinese with English abstract)

[CR5] Cheng J, Fan H, Li L et al (2018) Genome-wide identification and expression analyses of RPP13-like genes in barley. BioChip J 12:1–12

[CR6] Cuthbert PA, Somers DJ, Thomas J et al (2006) Fine mapping *Fhb1*, a major gene controlling fusarium head blight resistance in bread wheat (*Triticum aestivum* L). Theor Appl Genet 112:1465–1472 16518614 10.1007/s00122-006-0249-7

[CR7] Cuthbert PA, Somers DJ, Brulé-Babel A (2007) Mapping of Fhb2 on chromosome 6BS:a gene controlling Fusarium head blight field resistance in bread wheat(*Triticum aestivum* L). Theor Appl Genet 114:429–437 17091262 10.1007/s00122-006-0439-3

[CR8] Diethelm M, Schmolke M, Groth J et al (2014) Association of allelic variation in two NPR1-like genes with Fusarium head blight resistance in wheat. Mol Breeding 34:31–43

[CR9] Fu H, Zhao M, Xu J, et al (2020) Citron C-05 inhibits both the penetration and colonization of Xanthomonas citri subsp. citri to achieve resistance to citrus canker disease. Horticulture Research 7:58. 10.1038/s41438-020-0278-4 PMC7193574 32377349

[CR10] Gao CS, Kou XJ, Li HP et al (2013) Inverse effects of Arabidopsis NPR1 gene on Fusarium seedling blight and Fusarium head blight in transgenic wheat. Plant Pathol 62:383–392

[CR11] Gao X, Cheng B, GaoY, et al (2022) Genetic mapping of the stripe rust resistance gene from wheat Guixie 3. Journal of Triticeae Crops 42:948–957 (in Chinese with English abstract)

[CR12] Geng GD, Zhang SQ, Li ST et al (2009) Validation of the progenies from wide cross Israel wild emmer × Avena fatua L. var. Glabrata Pat by Molecular Markers Seed 28:35–37 (in Chinese with English abstract)

[CR13] Guo J, Zhang X, Hou Y et al (2015) High-density mapping of the major FHB resistance gene *Fhb7* derived from Thinopyrum ponticum and its pyramiding with *Fhb1* by marker-assisted selection. Theor Appl Genet 128:2301–2316 26220223 10.1007/s00122-015-2586-x

[CR14] Hao Q, Wang W, Han X et al (2018) Isochorismate-based salicylic acid biosynthesis confers basalresistance to Fusarium graminearum in barley. Mol Plant Pathol 19:1995–2010 29517854 10.1111/mpp.12675 PMC6638154

[CR15] He X, Singh PK, Schlang N et al (2014) Characterization of Chinese wheat germplasm for resistance to Fusarium head blight at CIMMYT. Mexico Euphytica 195:383–395

[CR16] He X, Lillemo M, Shi J et al (2016) QTL characterization of Fusarium head blight resistance in CIMMYT bread wheat line Soru#1. PLoS ONE 11:e0158052 27351632 10.1371/journal.pone.0158052 PMC4924825

[CR17] He Y, Ahmad D, Zhang X et al (2018) Genome-wide analysis of family-1UDP glycosyltransferases (UGT) and identification of UGT genes for FHB resistance in wheat (*Triticum aestivum* L). BMC Plant Biol 18:67 29673318 10.1186/s12870-018-1286-5 PMC5909277

[CR18] Huang C, Jiang YY, Li CG (2020) Occurrence, yield loss and dynamics of wheat diseases and insect pests in China from 1987 to 2018. Plant Prot 46:186–193 (in Chinese with English abstract)

[CR19] Jia G, Chen P, Qin G et al (2005) QTLs for Fusarium head blight response in a wheat DH population of Wangshuibai/Alondra‘s.’ Euphytica 146:183–191

[CR20] Kage U, Yogendra KN, Kushalappa AC (2017) *TaWRKY70* transcription factor in wheat QTL-2DL regulates downstream metabolite biosynthetic genes to resist Fusarium grami nearum infection spread within spike. Sci Rep 7:1–14 28198421 10.1038/srep42596 PMC5309853

[CR21] Li ML, Yan CQ, Wang YM et al (2010) Research advance of homology-based candidate gene method and its application in wild rice. Biotechnol Bull 21:126–129 (in Chinese with English abstract)

[CR22] Li RJ, Sheng ZY, Li XK et al (2020a) Genome-wide identification and expression analysis of NBS-LRR gene family in Setaria italica. Journal of Henan Agricultural Sciences 49:34–43 (in Chinese with English abstract)

[CR23] Li R, Wang L, Li Y et al (2020b) Knockout of SlNPR1 enhances tomato plants resistance against Botrytis cinerea by modulating ROS homeostasis and JA/ET signaling pathways. Physiol Plant 170:569–579 32840878 10.1111/ppl.13194

[CR24] Lin FY, Lu QX, Yang HY et al (2007) Advance on the molecular mechanism of the interaction between wheat and Fusarium graminearum. Journal of Triticeae Crops 27:934–938 (in Chinese with English abstract)

[CR25] Linkmeyer A, Hofer K, Rychlik M et al (2016) Influence of inoculum and climatic factors on the severity of Fusarium head blight in German spring and winter barley. Food Addit Contam Part A Chem Anal Control Expo Risk Assess 33:489–499 26679010 10.1080/19440049.2015.1133932

[CR26] Liu S, Zhang X, Pumphrey MO et al (2006) Complex microcolinearity among wheat, rice, and barley revealed by fine mapping of the genomic region harboring a major QTL for resistance to Fusarium head blight in wheat. Funct Integr Genomics 6:83–89 16270217 10.1007/s10142-005-0007-y

[CR27] Liu S, Hall MD, Griffey CA et al (2009) Meta-analysis of QTL associated with fusarium head blight resistance in wheat. Crop Sci 49:1955–1968

[CR28] Liu X, He Y, Yu LX et al (2020) Prokaryotic expression and protein purification of UDP-glucosyltransferase TaUGT6 gene in wheat (*Triticum aestivum L*). Molecular Plant Breeding 18:3470 (in Chinese with English abstract)

[CR29] Ma Z, Xie Q, Li G et al (2020) Germplasms, genetics and genomics for better control of disastrous wheat Fusarium head blight. Theor Appl Genet 133:1541–1568 31900498 10.1007/s00122-019-03525-8

[CR30] Ma S, Wang M, Wu J et al (2021) WheatOmics: a platform combining multiple omics data to accelerate functional genomics studies in wheat. Mol Plant 14:1965–1968 34715393 10.1016/j.molp.2021.10.006

[CR31] Makandar R, Essig JS, Schapaugh MA et al (2006) Genetically engineered resistance to Fusarium head blight in wheat by expression of Arabidopsis NPR1. Mol Plant Microbe Interact 19:123–129 16529374 10.1094/MPMI-19-0123

[CR32] Martin GB, Brommonschenkel SH, Chunwongse J et al (1993) Map based cloning of aprotein kinase gene conferring disease resistance in tomato. Science 262:1432–1436 7902614 10.1126/science.7902614

[CR33] McCartney CA, Brûlé-Babel AL, Fedak G et al (2016) Fusarium head blight resistance QTL in the spring wheat cross Kenyon/86ISMN 2137. Front Microbiol 7:1542 27790188 10.3389/fmicb.2016.01542 PMC5061752

[CR34] Mesterházy A (2006) Types and components of resistance to Fusarium head blight of wheat. Plant Breeding 114:377–386

[CR35] Narumanchi H, Goyal D, Emmadi N et al (2019) Mutation of a histidine-rich calcium-binding-protein gene in wheat confers resistance to Fusarium headblight. Nat Genet 51:1106–1112 31182810 10.1038/s41588-019-0426-7

[CR36] Prat N, Guilbert C, Prah U et al (2017) QTL mapping of Fusarium head blight resistance in three related durum wheat populations. Theor Appl Genet 130:13–27 27662843 10.1007/s00122-016-2785-0 PMC5215227

[CR37] Pritsch C, Muehlbauer GJ, Bushnell WR et al (2000) Fungal development and induction of defense response genes during early infection of wheat spikes by Fusarium graminearum. Mol Molecular Plant-Microbe Interactions 13:159–169 10659706 10.1094/MPMI.2000.13.2.159

[CR38] Pritsch C, Vance CP, Bushnell WR et al (2001) Systemic expression of defense response genes in wheat spikes as a response to Fusarium graminearum infection. Physiol Mol Plant Pathol 58:1–12

[CR39] Qi LL, Pumphrey MO, Friebe B et al (2008) Molecular cytogenetic characterization of alien introgressions with gene *Fhb3* for resistance to Fusarium head blight disease of wheat. Theor Appl Genet 117:1155–1166 18712343 10.1007/s00122-008-0853-9

[CR40] Rentel MC, Leonelli L, Dahlbeck D et al (2008) Recognition of the Hyaloperonospora parasitica effector ATR13 triggers resistance against oomycete, bacterial, and viral pathogens. Proc Natl Acad Sci USA 105:1091–1096 18198274 10.1073/pnas.0711215105 PMC2242713

[CR41] Somers DJ, Fedak G, Savard M et al (2003) Molecular mapping of novel genes controlling Fusarium head blight resistance and deoxynivalenol accumulation in spring wheat. Genome 46:555–564 12897863 10.1139/g03-033

[CR42] Song WY, Wang GL, Chen L et al (1995) A receptor kinase like protein encoded by the rice disease resistance gene, *Xa21*. Science 270:1804–1806 8525370 10.1126/science.270.5243.1804

[CR43] Steiner B, Buerstmayr M, Michel S et al (2017) Breeding strategies and advances in line selection for Fusarium head blight resistance in wheat. Tropical Plant Pathology 42:165–174

[CR44] Su P, Guo X, Fan Y et al (2018) Application of Brachypodium genotypes to the analysis of type II resistance to Fusarium head blight (FHB). Plant Sci 272:255–266 29807599 10.1016/j.plantsci.2018.04.015

[CR45] Su Z, Bernardo A, Tian B et al (2019) A deletion mutation in *TaHRC* confers *Fhb1* resistance to Fusarium head blight in wheat. Nat Genet 51:1099–1105 31182809 10.1038/s41588-019-0425-8

[CR46] Sun XH, Lu TG, Jia SR et al (2004) Genome-wide bioinformatics analysis of the rice receptor-like kinase family. Scientia Agricultura Sinica 37:322–327 (in Chinese with English abstract)

[CR47] Tamburic-Ilincic L, Rosa SB (2019) QTL mapping of Fusarium head blight and Septoria tritici blotch in an elite hard red winter wheat population. Mol Breeding 39:1–15

[CR48] Wang H, Sun S, Ge W et al (2020) Horizontal gene transfer of *Fhb7* from fungus underlies Fusarium head blight resistance in wheat. Science 368:5435 10.1126/science.aba5435 32273397

[CR49] Wang X, Li G, Jia H et al (2024) Breeding evaluation and precise mapping of *Fhb8* for Fusarium head blight resistance in wheat (*Triticum aestivum*). Plant Breeding 143:26–33

[CR50] Xiao S, Chen TQ, Gao X et al (2016) Resistance of Guixie 3 against wheat scab, strip rust and powdery mildew. Guizhou Agricultural Sciences 44:56–59 (in Chinese with English abstract)

[CR51] Xinyao, Ellis, Marc, et al (2016) Characterization of Fusarium head blight resistance in a CIMMYT synthetic-derived bread wheat line. Euphytica: International Journal of Plant Breeding 208:367–375.

[CR52] Xue S, Li G, Jia H et al (2010) Fine mapping *Fhb4*, a major QTL conditioning resistance to Fusarium infection in bread wheat (*Triticum aestivum L*). Theor Appl Genet 121:147–156 20198469 10.1007/s00122-010-1298-5

[CR53] Xue S, Xu F, Tang M et al (2011) Precise mapping *Fhb5*, a major QTL conditioning resistance to Fusarium infection in bread wheat (*Triticum aestivum L*). Theor Appl Genet 123:1055–1063 21739138 10.1007/s00122-011-1647-z

[CR54] Yang QZ, Yang PW, Wang Q et al (2001) Cloning and sequencing of disease resistance gene analogues in rice (*Oryza sativa L*). Chin J Rice Sci 15:241–247 (in Chinese with English abstract)

[CR55] Yang ZD, Ma X, Wu SW et al (2013) Cloning of NPR1-like genes and their response to Fusarium graminearum infection in wheat. Acta Agron Sin 39:1775–1782

[CR56] Yi TY, Xie BY, Zhang JX et al (2002) Application of plant resistance gene analogs in cloning and mapping resistance genes. Biotechnol Bull 2:16–20 (in Chinese with English abstract)

[CR57] Yu GH, Wang GP, Wang L et al (2015) Cloning of *PAL* genes and their response to FHB in wheat. Journal of Plant Genetic Resources 16:1055–1061 (in Chinese with English abstract)

[CR58] Yu G, Zhang X, Yao J et al (2017) Resistance against Fusarium head blight in transgenic wheat plants expressing the *ScNPR1* gene. J Phytopathol 165:223–231

[CR60] Zhang M, Zhang R, Yang J et al (2010) Identification of a new QTL for Fusarium head blight resistance in the wheat genotype “Wang shui-bai.” Mol Biol Rep 37:1031–1035 19757165 10.1007/s11033-009-9809-7

[CR61] Zhang Q, Axtman JE, Faris JD et al (2014) Identification and molecular mapping of quantitative trait loci for Fusarium head blight resistance in emmer and durum wheat using a single nucleotide polymorphism-based linkage map. Mol Breeding 34:1677–1687

[CR62] Zhang AM, Yang WL, Li X et al (2018) Current status and perspective on research against Fusarium head blight in wheat. Hereditas (Beijing) 40:858–873 (in Chinese with English abstract) 10.16288/j.yczz.18-252 30369469

[CR63] Zhang F, Zhang H, Liu J et al (2024) *Fhb9*, a major QTL for Fusarium head blight resistance improvement in wheat. J Integr Agric. 10.1016/j.jia.2024.03.045

[CR64] Zhao YT (2023) Studies of ascorbate peroxidase in response resistance to southern corn leaf. Henan Agricultural University (2) 10.27117/d.cnki.ghenu.2023.000363 (in Chinese with English abstract)

[CR65] Zhou T, Wang Y, Chen JQ et al (2004) Genome-wide identification of NBS genes in japonica rice reveals significant expansion of divergent non TIR NBS-LRR genes. Mol Genet Genomics 271:402–415 15014983 10.1007/s00438-004-0990-z

